# A Hearty Dose of Noncoding RNAs: The Imprinted *DLK1-DIO3* Locus in Cardiac Development and Disease

**DOI:** 10.3390/jcdd5030037

**Published:** 2018-07-10

**Authors:** Tiffany L. Dill, Francisco J. Naya

**Affiliations:** Department of Biology, Program in Cell and Molecular Biology, Boston University, Boston, MA 02215, USA; tiffdill@bu.edu

**Keywords:** cardiac, proliferation, hypertrophy, fibrosis, microRNA, long noncoding RNA, imprinting, epigenetics

## Abstract

The imprinted *Dlk1-Dio3* genomic region harbors a noncoding RNA cluster encoding over fifty microRNAs (miRNAs), three long noncoding RNAs (lncRNAs), and a small nucleolar RNA (snoRNA) gene array. These distinct noncoding RNAs (ncRNAs) are thought to arise from a single polycistronic transcript that is subsequently processed into individual ncRNAs, each with important roles in diverse cellular contexts. Considering these ncRNAs are derived from a polycistron, it is possible that some coordinately regulate discrete biological processes in the heart. Here, we provide a comprehensive summary of *Dlk1-Dio3* miRNAs and lncRNAs, as they are currently understood in the cellular and organ-level context of the cardiovascular system. Highlighted are expression profiles, mechanistic contributions, and functional roles of these ncRNAs in heart development and disease. Notably, a number of these ncRNAs are implicated in processes often perturbed in heart disease, including proliferation, differentiation, cell death, and fibrosis. However, most literature falls short of characterizing precise mechanisms for many of these ncRNAs, warranting further investigation. Taken together, the *Dlk1-Dio3* locus represents a largely unexplored noncoding regulator of cardiac homeostasis, harboring numerous ncRNAs that may serve as therapeutic targets for cardiovascular disease.

## 1. Introduction

Cardiovascular disease is associated with widespread perturbations in gene regulatory control that impact transcriptional, post-transcriptional, and epigenetic processes. Given the morphological and cellular complexity of the cardiovascular system, defining the gene regulatory networks required to form this vital physiological system is a formidable yet essential undertaking. This fundamental knowledge may yield insight into the genomic mechanisms through which insults, such as mutations, stress, and injury, trigger cellular abnormalities that ultimately drive disorders of the cardiovascular system.

Among various mediators of gene regulation, noncoding RNAs (ncRNAs) such as microRNAs (miRNAs) and long noncoding RNAs (lncRNAs) have emerged as core post-transcriptional and epigenetic regulators in the cardiovascular system [1,2,3,4,5]. During development, these classes of ncRNAs modulate gene programs controlling specification, proliferation, and differentiation in diverse cardiovascular cell types. The importance of ncRNAs in the cardiovascular system is exemplified by severe congenital defects that have emerged in gain/loss-of-function studies, their involvement in cardiac remodeling, and their dysregulated expression in a spectrum of cardiac disease phenotypes in human patients and animal model systems [6,7].

MiRNAs, one of the smallest classes of regulatory RNAs, inhibit gene expression through the degradation or translational block of protein-coding transcripts [8]. In the cardiovascular system, various miRNAs (miR-1/miR-133 clusters, miR-17–92 clusters) and the miRNA processing enzyme Dicer have been shown to play key regulatory roles in the differentiation of cardiomyocytes, smooth muscle cells, and neural crest derivatives in the developing heart [9,10,11,12,13,14]. MiRNAs also regulate cardiomyocyte proliferation and cardiac regeneration; for example, miR-590, miR-199a, and the miR-302–367 cluster can potently induce cardiomyocyte proliferation *in vitro* and *in vivo* [15,16]. In addition, the miR-15 family controls cell cycle withdrawal during early postnatal heart maturation in mice [17]. Given their central role in coordinating gene expression patterns that dictate cellular behavior within the cardiovascular system, these and many other miRNAs such as miR-208, miR-23a, and miR-29 have been implicated in modulating pathological remodeling in a spectrum of heart disease models [18,19,20].

LncRNAs, defined as polyadenylated RNAs greater than 200 nucleotides, exhibit complex stem-loop secondary structures, which along with their localization in the nucleus, cytoplasm, or both, enables them to regulate gene expression via multiple mechanisms [21,22]. Recent reports have described an important regulatory role for lncRNAs in cardiomyocyte specification and differentiation [7]. For example, the lncRNAs *Fendrr* and *Bvht* are important in cardiac development and cardiomyocyte lineage commitment, respectively [23,24]. Like their small ncRNA counterparts, differential expression of lncRNAs are observed in heart disease models, suggesting a role in pathological remodeling. Indeed the lncRNAs *Mhrt, Chaer, MIAT*, and *CARL*, to name a few, which are dysregulated in cardiac disease, confer epigenetic perturbations by modulating chromatin factor activity, or augment gene expression by sequestering miRNAs from their target transcripts [25,26,27,28].

Numerous reviews have described in great detail the function of aforementioned miRNAs and lncRNAs in cardiac development and disease. Whereas most miRNAs and lncRNAs identified to date are encoded as a single inter- or intragenic genes, or in some instances, expressed from a small cluster of several related RNAs, the mammalian *Dlk1-Dio3* ncRNA locus is unusually massive: a >200 kilobase (kb) mega-cluster of distinct regulatory RNA functional categories. Specifically, more than 50 miRNAs, several lncRNAs, and a tandemly repeated array of C/D-box snoRNAs (small nucleolar RNAs) are expressed from this locus [29,30]. The *Dlk1-Dio3* locus has been investigated extensively as a paradigm to understand the epigenetic mechanisms of genomic imprinting [31,32,33,34]. In this review, we highlight recent evidence that various ncRNAs expressed from this large, imprinted locus have key regulatory functions in developmental and disease-related processes pertaining to the cardiovascular system. We first explore a large body of findings that implicate many *Dlk1-Dio3* miRNAs in cardiomyocyte proliferation and differentiation, heart development, and cardiac disease pathways. Then, we turn our attention toward *Gtl2*, a locus lncRNA with recently established roles in cardiac fibrosis and the vasculature. By highlighting the individual functions of the various *Dlk1-Dio3* locus ncRNAs, a broader picture may emerge regarding a dynamic role for this remarkably complex locus in the cardiovascular system.

### 1.1. The Dlk-Dio3 ncRNA Locus: Gene Organization, Imprinting and Human Disease

**Figure 1:**
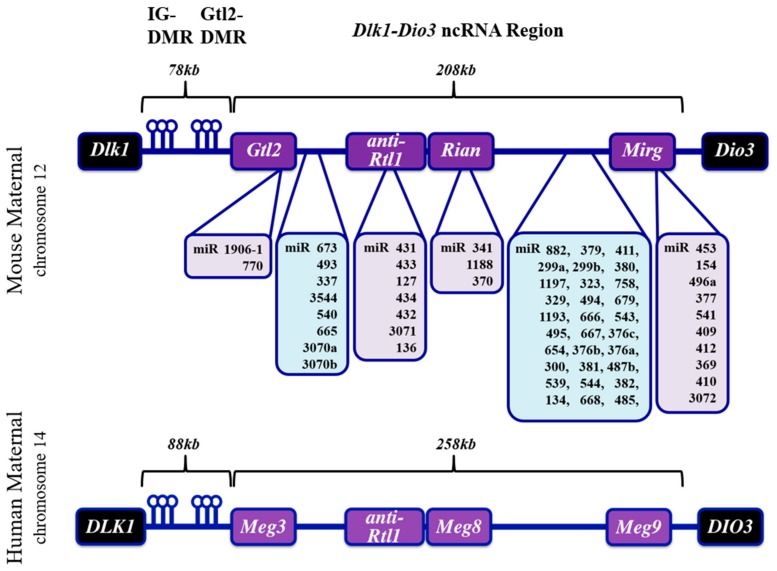
Schematic of *Dlk1-Dio3* ncRNA locus features. The mouse *Dlk1-Dio3* ncRNA locus (top panel) harbors sixty-one miRNAs and three lncRNA genes. Several miRNAs (shaded in light purple) reside within *Gtl2*, *anti-Rtl1*, *Rian*, and *Mirg* coding regions (shaded in light purple), whereas many miRNAs (shaded in light blue) exist between the aforementioned coding regions. The human *DLK1-DIO3* ncRNA locus (bottom panel) harbors fifty-three miRNAs, which are largely present in the same regions as the mouse locus. Both loci are regulated by a 5′ intergenic differentially methylated region (IG-DMR), which is upstream of the *Gtl2*-DMR. While most lncRNAs are homologous between mouse and human gene annotations, it is important to note the mouse *Mirg* lncRNA harbors miRNAs, whereas the human *Meg9* lncRNA does not. Instead, *Meg9* resides downstream of all annotated human locus miRNAs. NcRNAs are expressed from the non-methylated maternal allele, whereas methylation of the IG-DMR in the paternal chromosome (not depicted) prevents expression of ncRNAs. Protein-coding genes (shaded in black) are expressed predominantly from the paternal allele [33].

The mammalian *Dlk1-Dio3* ncRNA locus resides on chromosomes 12 and 14 in mice and humans, respectively, and derives its name from the protein-coding genes that flank the ncRNA sequences (Figure 1). The organization of the locus is conserved in all mammals. The *Dlk1* (Delta-like homolog 1) gene is located upstream of the ncRNA region and codes for a protein involved in the Notch signaling pathway [35]. Located at the 3′-end of the imprinted region is the *Dio3* (type III iodothyronine deiodinase) gene which functions in thyroid hormone signaling [36]. Between the murine *Dlk1* and *Dio3* genes resides the >200 kb ncRNA mega-cluster: beginning with the lncRNA *Gtl2* (*MEG3* in humans) at the 5′ end and continuing through miR-3077 at the 3′ end [30]. The *Gtl2* lncRNA is the most 5′ lncRNA transcript, exists in multiple splice variants, and interacts with chromatin remodeling proteins [37]. *Anti-Rtl1*, which harbors several miRNAs, is located immediately downstream of the *Gtl2* gene. This region is followed by *Rian*, an annotated lncRNA which has no known function apart from harboring a tandemly repeated array of the C/D-box snoRNA family and three miRNAs. At the 3′ end of the locus is *Mirg*, which encodes a cluster of dozens of miRNAs (miR-379–410) that appear to fall into discrete subfamilies based on sequence similarities [29]. Moreover, although *Mirg* has no known lncRNA function, the transcript exhibits diverse tissue-specific expression in mouse embryogenesis [38].

Between the *Dlk1* gene and the transcription start site of the ncRNA cluster are two intergenic (IG), differentially DNA-methylated regions (DMR): the IG-DMR and *Gtl*2-DMR [32]. The IG-DMR is located approximately 20 kb upstream of *Gtl2*, and its methylation state has been shown to dictate mono-allellic expression of the locus (i.e., protein-coding genes are exclusively expressed from the methylated paternal allele, whereas ncRNAs are expressed exclusively from the maternal allele). The *Gtl2*-DMR overlaps the proximal promoter and methylated CpG islands; in contrast, the IG-DMR is largely homomethylated, but it is currently unclear whether this epigenetic modification imparts any function [39]. A schematic depicting the organization of *Dlk1-Dio3* locus ncRNAs is provided in Figure 1. 

In humans, imprinting abnormalities at the *Dlk1-Dio3* locus have been linked to syndromes of impaired fetal development and postnatal growth. Humans with uniparental disomy, that is, duplicate copies of either maternal or paternal chromosome 14q32 (MatUPD14 or PatUPD14), display severe growth retardation, skeletal malformations, and metabolic deficiencies [40]. Two known examples of chromosome 14q32 uniparental disomy are Temple and Kagami-Ogata syndromes [41,42]. Among the aforementioned clinical features in these patients, muscle hypotonia is a prominent symptom, and suggests a critical function for this locus in muscle development. Though not a prevalent feature, cardiac abnormalities such as septal defects and cardiomegaly have been described in a small number of patients with chromosome 14 uniparental disomy [43,44]. Mouse models of uniparental disomy of chromosome 14 (or mouse chromosome 12) display similar phenotypes to the human syndromes. Both paternal disomy (PatUPD12) and maternal disomy (MatUPD12) in mice display placental, bone, and skeletal muscle defects [45].

It is not entirely clear how the multiple ncRNAs are expressed from the *Dlk1-Dio3* locus, since there are conflicting reports describing their transcriptional regulation. Multiple lines of evidence suggest mature *Dlk-Dio3* ncRNAs are derived from post-transcriptional processing of a single, polycistronic transcript, synthesized from a promoter directly upstream of the first exon of the *Gtl2* lncRNA. Supporting this notion, coordinate dysregulation of *Dlk1-Dio3* miRNAs and lncRNAs has been reported in several experimental models indicating a common transcriptional start site for these ncRNAs [46,47,48,49,50,51]. However, other studies have reported discrete enhancers neighboring miRNA clusters within the ncRNA locus. For example, the miR-379–410 cluster is regulated by a MEF2-dependent enhancer immediately upstream of miR-379 [52]. Also, enhancers regulated by the estrogen receptor have been described upstream of the miR-433–127 cluster [53]. Regardless of the mechanism by which these ncRNAs are transcribed, while some of the miRNAs appear to target components belonging to a given pathway, many of the miRNAs expressed from this locus have distinct biological targets. Thus, much remains to be resolved regarding the regulation and activity of the *Dlk1-Dio3* ncRNA locus.

### 1.2. The Dlk1-Dio3 ncRNA Locus in Striated Muscle

There is considerable evidence demonstrating an essential requirement for the ncRNAs in the *Dlk1-Dio3* locus in mammalian striated muscle. In mice, a 10 kb deletion encompassing the *Gtl2* lncRNA coding region (exons 1–5) and ~300 bp of the proximal promoter resulted in pronounced skeletal muscle defects in late fetal development [47]. Consistent with the genomic imprinting status and maternal expression of the ncRNAs, heterozygous mice with maternal inheritance of the deletion displayed the muscle phenotype whereas paternal inheritance did not affect growth or muscle development. Curiously, an independent line of knockout mice harboring a similar deletion as Zhou *et al.* described postnatal growth retardation with pulmonary and hepatic defects, but skeletal muscle defects were not reported [46]. It is worthwhile to note that both deletions caused the downregulation of all ncRNAs in the locus, reinforcing the notion of coordinate regulation.

Additional mouse models have linked the *Dlk1-Dio3* ncRNA locus to skeletal muscle phenotypes. Homozygous mice harboring a deletion of the miR-379–544 cluster resulted in skeletal muscle hypertrophy, a phenotype likely attributable to altered regulation of the neighboring *Dlk1* gene by one or more of the miRNAs embedded within this cluster [54]. In contrast, deletion of the miR-379–410 cluster, which removed additional miRNAs in the *Mirg* region downstream of miR-544, resulted in liver metabolic deficiencies and neonatal lethality. Although the authors measured significant downregulation of miRNAs in both heart and skeletal muscle, no further characterization was described [55]. Our group previously demonstrated that the *Dlk1-Dio3* ncRNA locus is directly regulated by the MEF2 transcription factor and that this pathway is involved in muscle differentiation and regeneration [50]. Consistent with these findings, the MEF2-*Dlk1-Dio3* pathway was recently demonstrated to function in muscle stem cell quiescence, metabolism, and differentiation [56,57]. Furthermore, genome-wide profiling of skeletal muscle in aged mice revealed coordinate downregulation of eight *Dlk1-Dio3* miRNAs [58]. Finally, the *Dlk1-Dio3* locus has been implicated in skeletal muscle hypertrophy. *Callipyge* sheep harbor a point mutation between the *Dlk1* gene and the beginning of the ncRNA region (*Gtl2/Meg3*), and when inherited from the paternal allele, these animals develop muscle hypertrophy. This mutation presumably affects the activity of a distal enhancer, which alters protein-coding DLK1 and RTL/PEG11 genes expressed from the paternal allele [59,60]. There is also evidence that ncRNA expression is affected in *callipyge* sheep [61]. Taken together, it appears that the *Dlk1-Dio3* locus and a subset of ncRNAs have biological relevance in skeletal muscle, although it remains unclear whether cardiac defects exist in *callipyge* sheep or any of the aforementioned mouse mutants.

## 2. MicroRNAs Expressed from the *Dlk1-Dio3* Locus in Cardiac Differentiation and Development

Despite the apparent lack of a cardiac phenotype in the various *Dlk1-Dio3* mutant mouse lines, either because this organ has only been analyzed superficially or these mutations do not specifically cause cardiac abnormalities, there are numerous reports describing expression profiling, dysregulation, and functional roles for *Dlk1-Dio3* ncRNAs in the cardiovascular system. In recent years, links between the *Dkl-Dio3* locus and cardiac developmental processes, including proliferation and differentiation, have notably increased. Below, we summarize those *Dlk1-Dio3* miRNAs and their connections to specific cardiac processes, as they are currently understood.

### 2.1. Cardiomyocyte and Endothelial Progenitors

**Figure 2:**
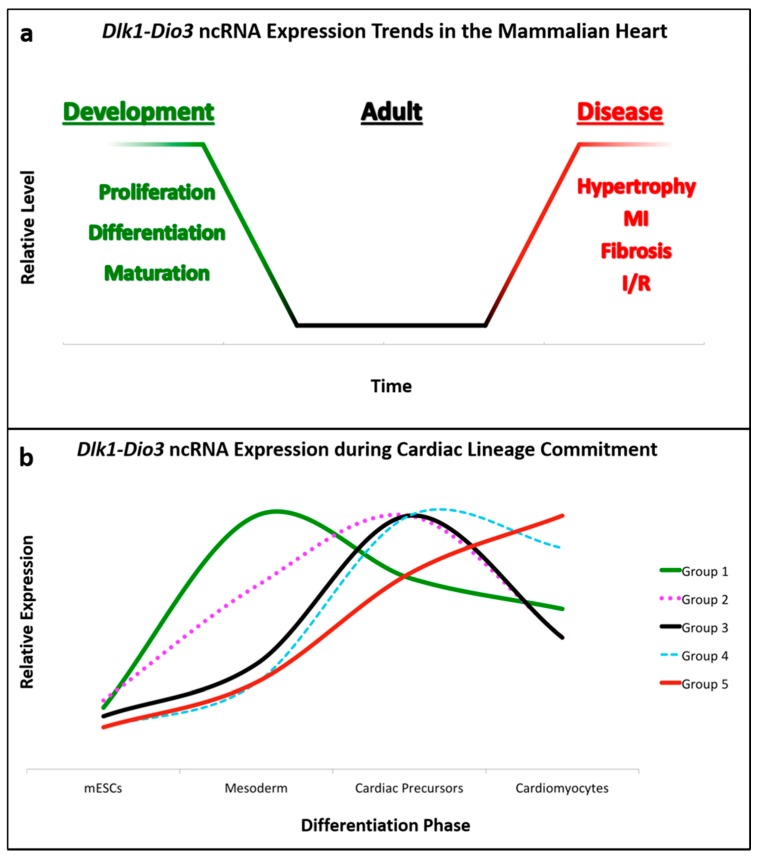
*Dlk1-Dio3* ncRNA Expression in the Heart and Cardiomyocyte Differentiation. (**a**) General trends of locus ncRNA expression over time: Many ncRNAs are expressed at their highest levels during fetal and early-postnatal cardiac development, and are gradually downregulated to low basal levels during adulthood. However, disease or stress triggers marked upregulation of numerous locus ncRNAs. (**b**) Locus ncRNAs are dynamically expressed throughout phases of cardiomyocyte lineage commitment and differentiation. This plot was generated using supplementary data from Wamstad et al. [62], which analyzed the transcriptome during cardiomyocyte-directed differentiation. To generate the plot, raw values of individual ncRNAs were normalized to their maximal expression, and subsequently grouped by the differentiation phase at which maximal ncRNA expression was reached. The relative levels conveyed represent the average of all ncRNAs within a given group, and are comprised as follows: Group 1 = miR-1906, -770, -493, -337, -540, -665, -432, -1188, -882, -299, -380-5p, -323-5p, -758, -679, -666, -654, -544, -485, -453, -412, and -369-5p; Group 2 = miR-673-5p, -341, -370, -494, -667^+^, -376b^+^, -300^+^, -and 541; Group 3 = miR-431^+^, -127^+^, -434-5p^+^, -1197, -323-3p^++^, -1193^++^, -543^++^, -495^++^, -539, -134^+^, -668, -496, -409-3p, and -410^+^; Group 4 = Gtl2, miR-379^+^, -411^+^, -376c^++^, -376a^+^, -381^+^, and -382^+^; Group 5 = miR-433^+^, -136^++^, -380-3p, -487b^+^, -154^+^, -377^+^, -409-5p^+^, and -369-3p^++^. To account for levels, ncRNAs annotated with “+” indicate measurements over 100 reads, and “++” for over 1000 reads. Although groups 2, 3, and 4 all peak in cardiac precursors, these ncRNAs were subdivided into groups based on striking differences in expression levels during the mesoderm (early) and cardiomyocyte (later) phases. These ncRNA patterns illustrate the dynamic regulation of the locus. Consistent with the high levels reported in the fetal and postnatal heart, locus ncRNAs are enriched in later phases of cardiac differentiation.

Temporal expression of *Dlk1-Dio3* ncRNAs has been documented in directed cardiomyocyte differentiation from mouse embryonic stem cells. Although not the primary focus of the investigation, transcriptomic analysis revealed that ncRNA expression from this locus is highly upregulated in the transition from pluripotent stem cells to differentiated cardiomyocytes [62]. Interestingly, sub-groups of *Dlk1-Dio3* miRNAs displayed differences in their temporal expression during phases of cardiomyocyte differentiation, which are summarized in Figure 2b. These observations suggest a function for one or more of *Dlk1-Dio3* ncRNAs in cardiac lineage commitment, and may underscore dynamic roles for individual ncRNAs in heart development.

In mice, miR-300 has been shown to suppress cardiomyocyte progenitor differentiation *in vitro*. Specifically, miR-300 overexpression impaired cardiomyocyte differentiation marker expression and reduced the population of Sca1+ cardiac progenitor cells (CPCs) isolated from adult mouse hearts. Interestingly, overexpression of Bmi1, a member of the Polycomb Repressor Complex 1 (PRC1), appeared to significantly upregulate miR-300 in CPCs, but the treatment did not influence other miRNAs in the *Dlk1-Dio3* locus [63].

Survival of CPCs from fetal human hearts is modulated by another distinct locus miRNA, miR-134. Overexpression of miR-134 in CPCs reduced proliferation, whereas inhibition enhanced proliferation. Numerous cell cycle genes, including cyclins A, B, and E, and CDK4, displayed dysregulated expression in these experiments. Moreover, miR-134 regulated cell cycle activity by targeting Meis2, a TALE homeobox transcription factor that regulates CPC proliferation and differentiation. Terminal differentiation of these progenitor cells was not affected by overexpression or inhibition of miR-134, but inhibition of this miRNA reduced levels of the cardiac transcription factors MEF2C, GATA4, and NKX2.5 [64].

Expression of the *Dlk1-Dio3* miRNAs mir-495 and miR-543 was shown to correlate with the commitment of endothelial cells from mesodermal precursors derived from human induced pluripotent stem cells (iPSCs). Specifically, miR-495 and miR-543 were found to be significantly downregulated in CD31+ endothelial cells compared to non-endothelial cells (CD31-) and undifferentiated human iPSCs. Consistent with the expression analysis, stable human iPSC lines harboring antisense oligonucleotide against miR-495 enhanced endothelial cell differentiation. Moreover, miR-495 inhibition promoted angiogenesis (tube formation and migration) from human iPSC-endothelial cells *in vitro*. One target in this process is vascular endothelial zinc finger 1 (VEZF1), a regulator of endothelial cell differentiation and angiogenesis [65].

When considering temporal expression and several functional studies, it appears many *Dlk1-Dio3* ncRNAs are involved with the commitment of mesoderm to a diverse subset of specialized cardiac cell lineages. The opposing roles of some *Dlk1-Dio3* miRNAs in these contexts may reflect the versatility required for a single genetic locus to allocate diverse cell fates in early cardiac formation. Indeed, discrete subsets of miRNAs exhibit distinct expression patterns, and as demonstrated with Bmi1 manipulations, it is evident that mechanisms exist to enrich specific locus miRNAs over others. It therefore seems likely that transcriptional regulators interact with the locus polycistron during differentiation, dictating which locus miRNAs are primed to favor—or suppress—a particular cell fate.

### 2.2. Cardiac Structures

Morphogenesis of specialized cardiac regions, such as the chambers, conduction system, and valves, requires precise spatio-temporal regulation of gene expression patterns that govern development of these structures. MiRNAs play an important role in fine-tuning such gene expression. To identify miRNAs potentially involved with development and/or function of specific cardiac structures, comparative transcriptomic analyses were performed on discrete cardiac regions from three mammalian species: rat, dog, and monkey. This ultimately identified miRNAs enriched in specific regions of the adult heart and conserved across the three mammalian species. One miRNA from the *Dlk1-Dio3* locus, miR-127, was found to be enriched in cardiac valves in rat, dog, and monkey [66]. It is surprising that this particular miRNA appears to be the only *Dlk1-Dio3* ncRNA enriched in this cardiac structure, suggesting region-specific post-transcriptional regulation of these miRNAs in the heart.

Ventricular septal defects are among the most common congenital cardiac abnormalities in humans [67]. A microarray screen to identify circulating miRNAs in ventricular-septal defect patients revealed *Dlk1-Dio3* locus miRNAs-379, -654, -487b, -409, and -433 are significantly downregulated in plasma from these patients. Of these, all but miR-654 were independently validated using RT-PCR, and the authors used miR-targeting algorithms to model how miR-433 could regulate septal development: targeting NOTCH1 and/or the GATA3 transcription factor [68]. It is difficult to discern whether these circulating miRNAs are directly involved in ventricular septal defects, or a secondary consequence of this congenital disease. Along with the expression of miR-127 in the cardiac valves, these observations suggest a potential function for the *Dlk1-Dio3* locus in cardiac morphogenesis, and warrant further investigation of these ncRNAs for pathophysiological roles in defective cardiac structures.

### 2.3. Cardiomyocyte Proliferation and Apoptosis

Understanding the mechanisms of cell cycle regulation in cardiomyocytes is a major driving force in cardiac regenerative medicine given the implications of repairing damaged tissue through myocyte proliferation. Studies have demonstrated that certain *Dlk1-Dio3* miRNAs are capable of stimulating proliferation of post-mitotic neonatal cardiomyocytes. Although not the major focus of the study, a functional screen for pro-proliferative miRNAs in cardiomyocytes listed several *Dlk1-Dio3* miRNAs, miR-411, -380, -495, -539, -668, -154, and -410 as capable of inducing DNA synthesis and cytokinesis in neonatal mouse and rat cardiomyocytes [15]. We have shown that overexpression of miR-410 or miR-495 induces robust proliferation of neonatal rat ventricular myocytes (NRVMs) *in vitro*. Both of these miRNAs directly targeted and inhibited Cited2, a transcriptional coactivator, resulting in the upregulation of pro-proliferative genes and inhibition of apoptotic genes [69].

MiRNAs that stimulate proliferation of post-mitotic cardiomyocytes may reflect their cell cycle regulatory activity in cardiac development. Along these lines, miR-410 and miR-495 and other miRNAs in the *Dlk1-Dio3* locus such as miR-337 and miR-668 are expressed at higher levels in the neonatal mouse heart compared to the adult and first week post-natally, respectively [69,70]. Similarly, a miRNA screen comparing heart tissue before and after birth in sheep revealed higher expression of *Dlk1-Dio3* miRNAs (-493, -127, -432, -379, -411, -380, -329, -543, -487b, -382, -485, -154, -409, and -410) in late fetal cardiac development compared to the post-natal heart [51]. The downregulation of *Dlk1-Dio3* miRNAs coincides with a developmental timepoint where the regenerative capacity of the murine heart is lost, a consequence of cardiomyocyte cell cycle withdrawal.

Cardiomyocyte survival is also dependent on the *Dlk1-Dio3* miRNA miR-377. The drug cyclosporin A (CsA) inhibits calcineurin, can block cytochrome c release, and also induces cardiomyocyte apoptosis. In NRVMs treated with CsA, miR-377 was the most upregulated miRNA. It was further characterized that miR-377 is a downstream mediator of CsA-induced cardiomyocyte apoptosis. Inhibition of miR-377 in CsA-treated NRVMs attenuated apoptosis, whereas miR-377 overexpression alone promoted cardiomyocyte apoptosis [71]. These results indicate a pro-apoptotic role for miR-377 in neonatal cardiomyocytes *in vitro*.

Data reported to date clearly indicate a function for *Dlk1-Dio3* miRNAs in cardiomyocyte proliferation, whereas miR-377 functions in an entirely distinct manner: facilitating death. Continued investigation into transcriptional regulation of these miRNAs, as well as their therapeutic potential, is merited given their ability to modulate post-mitotic cardiomyocyte behaviors—specifically cardiomyocyte proliferation, which is extremely limited in the adult heart, and apoptotic cell death, which contributes to functional loss of heart muscle during disease.

## 3. MicroRNAs Expressed from the *Dlk1-Dio3* Locus in Cardiovascular Pathology

In most tissues including the heart, post-natal expression of *Dlk1-Dio3* ncRNAs is substantially downregulated. However, disease or exposure to various cardiac stressors upregulates these ncRNAs. Below, we summarize current research that implicates locus miRNAs in pathological remodeling of the heart, and highlight specific functions that may contribute to (or attenuate) heart disease.

### 3.1. Cardiac Hypertrophy

Hypertrophic myocyte growth is a hallmark of pathological cardiac remodeling, observed in numerous heart disease modalities. Because chronic hypertrophy inflicts deleterious functional consequences, its signaling and transcriptional mechanisms have been studied exhaustively. It is now firmly established that miRNAs impart important regulatory roles in pathological cardiac remodeling. Several miRNAs in the *Dlk1-Dio3* locus have been implicated in cardiac hypertrophy, and their dysregulated expression has been described in various cardiac disease models associated with hypertrophy.

Our group reported that both miR-410 and miR-495 are upregulated in angiotensin II (Ang II)-induced hypertrophy *in vivo* and in neonatal rat ventricular myocytes (NRVMs) treated with phenylephrine (PE). AntimiRs that specifically inhibit miR-410 or miR-495 were shown to attenuate PE-induced hypertrophy in NRVMs. Moreover, we showed that the MEF2-dependent *Gtl2* proximal promoter region is active in NRVMs, and this activity is amplified in response to PE, suggesting the ncRNA locus is sensitive to hypertrophic signals [72]. 

A microarray that surveyed miRNAs in serum from cats with spontaneous hypertrophic cardiomyopathy indicated that locus ncRNA miR-381 was upregulated. It was noted that *in silico* analyses revealed miR-381 has more predicted cardiac mRNA targets than most other miRNAs highlighted in the study, suggesting a pervasive role for this miRNA in the heart [73]. MiR-154 was also found to be upregulated in pressure overload and in hypertrophic cardiomyopathy in humans. Its inhibition in mice subjected to transverse aortic constriction (TAC) attenuated dysfunction and fibrosis, and it was determined that miR-154 directly targets p14, a cell cycle inhibitor [74]. Collectively, these data reveal the pro-hypertrophic activity attributed to a subset of *Dlk1-Dio3* miRNAs.

Surprisingly, while the above *Dlk1-Dio3* miRNAs have been shown to be upregulated in hypertrophic NRVMs, a subset of locus miRNAs display pathological downregulation, and unsuprisingly, these downregulated locus miRNAs functionally mitigate hypertrophy. For example, miR-485 was downregulated in PE-treated NRVMs, and unlike the aforementioned miRNAs, its overexpression attenuated cardiac hypertrophy *in vivo* [75]. MiR-541 has also been shown to be downregulated in hypertrophic cardiomyocytes induced by Ang II treatment. Consistent with these observations, transgenic mice that overexpress miR-541 in the heart displayed reduced hypertrophy in response to chronic Ang II treatment. This same study also described a promoter upstream of miR-541 and showed that it is negatively regulated by the MITF transcription factor [76].

Taken together, the dysregulation of the aforementioned miRNAs indicates a central role for these regulatory RNAs in facilitating cardiomyocyte hypertrophy. Moreover, the observation that some miRNAs display opposing patterns of dysregulation and antagonistic modulatory effects suggests complex post-transcriptional regulation of these transcripts, which appear to ultimately favor hypertrophic signaling in disease. If specific post-transcriptional mechanisms that dictate the balance between pro- and anti-hypertrophic *Dlk1-Dio3* miRNAs were to be identified, they would constitute an appealing therapeutic target for modulating locus activity in hypertrophic remodeling.

### 3.2. Myocardial Infarction

Myocardial infarction (MI) stems from reduced blood flow or complete blockage in vessels surrounding the heart, which ultimately results in extensive cardiomyocyte death and increased fibrosis. While the cellular pathophysiology of MI has been well documented, the post-transcriptional and epigenetic pathways that promote adverse myocardial remodeling remain poorly understood. Dysregulation of ncRNAs including the *Dlk1-Dio3* ncRNA locus has been documented in animal models of MI, with a handful being functionally characterized.

One study examining miRNA profiles of mice subjected to MI revealed that nearly one-third of dysregulated miRNAs in this disease model belonged to the *Dlk1-Dio3* locus, and all were found to be significantly upregulated though to varying levels [77]. In agreement with these observations, we found miR-410 and miR-495 to be dynamically upregulated in the infarcted mouse heart. The extent of upregulation in the infarct zone, where fibroblasts are abundant and myocytes are sparse, was considerably greater in the injured region relative to the remote area [72]. This may indicate roles for these specific miRNAs in fibroblast proliferation or activation, rather than surrounding cardiac muscle.

Additional MI studies independently demonstrate dysregulation of the *Dlk1-Dio3* miRNAs miR-539, miR-433, miR-377, and miR-370. One such study focused on the potential metabolic role of miR-539 in MI. This *Dlk1-Dio3* miRNA was shown to be upregulated in MI and to directly regulate O-linked GlcNAcase (OGA), a metabolic enzyme reduced in MI, thereby resulting in a pathological increase in O-linked GlcNAcylation of proteins [78]. Moreover, miR-539 contributes to MI-induced myocyte apoptosis and targets the mitochondrial prohibitin complex subunit PHB2 [28]. Another MI study performed in mice found miR-433 to be significantly upregulated in this cardiac injury model. Inhibition of miR-433 significantly reduced fibrosis, and preserved left ventricular ejection fraction and fractional shortening. In this same study, overexpression of miR-433 promoted cardiac fibroblast proliferation, and promoted pathological differentiation of fibroblasts into myofibroblasts [79]. Finally, miR-370 and miR-323 were upregulated in myocardial tissue from pigs subjected to coronary microembolization [80]. miR-370 was also expressed at significantly high levels in plasma from patients with coronary artery disease [81]. 

Taken together, we conclude that a large subset of *Dlk1-Dio3* miRNAs are upregulated in MI models, and some have the capacity to functionally drive pathological cell behaviors. It seems a variety of locus miRNAs could serve as biomarkers, and targeting a subset of these miRNAs could mitigate the deleterious effects of fibrosis in cardiac injury.

### 3.3. Cardiac Fibroblasts

Many heart diseases, including MI, are associated with increased fibrosis. Fibrosis stems from cardiac fibroblast activation, proliferation, and increased extracellular matrix (ECM) production. In heart disease, fibrotic regions replace dead myocytes, but fail to restore heart function, which ultimately burdens surviving cardiomyocytes. Thus, understanding the mechanisms of fibroblast activity can help identify targets that mitigate the area of fibrosis and preserve contractile function in spite of disease. 

To date, only two miRNAs expressed from the *Dlk1-Dio3* locus have been explicitly characterized in cardiac fibrosis, each with opposing effects on fibroblast behavior. MiR-154 is dysregulated in pressure overloaded mice, and overexpression resulted in increased cardiac fibroblast proliferation via Wingless-related integrated site (WNT) signaling, whereas its inhibition attenuated fibrosis in the heart [82,83]. In contrast, miR-495 overexpression was found to inhibit myofibroblast inflammation, differentiation, and excess ECM accumulation in human cardiac fibroblasts exposed to high glucose-induced inflammation. MiR-495-mediated effects appear to be mediated via modulation of the NF-kB and TGFβ pathways [84]. Although these are only two miRNAs that have been characterized in cardiac fibroblasts, it strikingly illustrates how *Dlk1-Dio3* locus miRNAs can mediate opposing cellular behaviours in the same cell context.

### 3.4. Ischemia/Reperfusion

Cardiac ischemia, i.e., reduced blood supply to the heart, precedes overt myocardial infarction. Reperfusion, i.e., reoxygenation of the heart, causes extensive tissue damage and oxidative stress, particularly in the vasculature. Several ischemia/reperfusion (I/R) models in mice have shown dysregulated *Dlk1-Dio3* miRNA expression, suggesting these ncRNAs are involved in cardiac remodeling associated with this surgically-induced injury process.

Inhibition of the *Dlk1-Dio3* miRNAs miR-329, miR-487b, miR-494, miR-495 promoted neovascularization after ischemia [85]. Similarly, inhibition of miR-377 in CD34+ cells which were transplanted in I/R-induced mice promoted neovascularization and reduced fibrosis post-I/R injury. Serine/threonine kinase 35 was shown to be regulated by miR-377 [86]. Consistent with this finding, miR-377 was downregulated in hypoxia-treated mesenchymal stem cells (MSCs), and injection of miR-377-deficient MSCs into the infarcted rat heart reduced fibrosis and improved cardiac function [87]. MiR-410 was found to be upregulated in cardiac I/R injury in mice and in hypoxia/reoxygenation stimulated human adult cardiac myocytes, and its inhibition attenuated mitophagy in I/R [88]. Contrary to the aforementioned findings, miR-494 was shown to be downregulated in I/R mice, and transgenic mice overexpressing miR-494 improved cardiac function and reduced infarct size [89]. 

Collectively, these studies describe functionally distinct *Dlk1-Dio3* locus miRNAs upregulated in I/R, which broadly facilitates pathological remodeling. Similar to other instances, exceptions to the overall trend in coordinate locus-wide expression may reflect complex post-transcriptional modulation. Intriguingly, I/R appears to trigger pathological processing of the locus polycistron, favoring biologically detrimental miRNAs over functionally antagonistic ones. 

### 3.5. Circulating miRNAs in Cardiovascular Disease

Many miRNAs circulate in blood plasma, and given their potential utility as non-invasive biomarkers, much work has been directed towards identifying circulating miRNAs that change with cardiovascular disease [90]. Several *Dlk1-Dio3* miRNAs have been identified in studies surveying circulating RNAs from the sera of patients with congestive heart failure and other cardiovascular complications. A significant increase in miR-299 and mir-665 levels has also been detected in serum from human patients with chronic heart failure [91,92]. Another study demonstrated that miR-665 and miR-494 are downregulated and upregulated, respectively, in heart failure. This opposite effect on expression was thought to regulate the expression of cannabinoid receptor subtypes [93].

The *Dlk1-Dio3* miRNAs miR-134 and miR-380 have been found to be enriched in plasma from patients with acute pulmonary embolism and acute myocardial infarction [38,94,95].

Upregulation of miR-494 and miR-495 expression are found in patients with arrhythmogenic right ventricular cardiomyopathy (ARVC) and dogs with myxomatous mitral valve disease, respectively [96,97]. In other cardiovascular diseases, reduced levels of *Dlk1-Dio3* miRNAs have been reported. For example, miR-382 and miR-432 are downregulated in plasma from patients with aortic stenosis and atrial fibrillation, respectively [98,99]. Although the physiological contributions of these circulating *Dlk1-Dio3* locus miRNAs remain unclear, their dynamic expression may have utility as a panel of biomarkers for cardiovascular disease.

The opposing effects of the aforementioned miRNAs in a spectrum of cardiac disease models, where *Dlk1-Dio3* locus miRNAs function antagonistically, reinforces the notion of a multifaceted role for this locus in modulating disease pathways. A summary of miRNA functions in development and disease can be found in Table 1.

## 4. Long noncoding RNAs Expressed from the *Dlk1-Dio3* Locus in Cardiac Development and Disease

At the time of this review, the lncRNA *Gtl2* (*Meg3*) remains the only functionally characterized lncRNA in the *Dlk1-Dio3* locus. In non-cardiac cells, *Gtl2/Meg3* has been hypothesized to function as a tumor suppressor given its reduced expression in a variety of tumors including pituitary adenomas [24]. Overexpression of *Gtl2/Meg3* in various tumor cell lines caused a reduction in proliferation, lending support to its purported tumor suppressor function. Moreover, *Gtl2/Meg3* has been shown to induce expression levels of the p53 tumor suppressor and enhance its transcriptional activity [105]. It is not yet known whether *Gtl2/Meg3* lncRNA functions in an anti-proliferative capacity in cardiomyocytes. In embryonic stem cells, *Gtl2/Meg3* interacts with the polycomb repressive complex (PRC2). The ability to regulate this chromatin modifier enzyme suggests that *Gtl2/Meg3* plays an important role in pluripotency and epigenetics [37,106,107].

Apart from its reported temporal expression in directed cardiomyocyte differentiation, a function for *Gtl2/Meg3* has not been defined in cardiac development. Nevertheless, functions for *Gtl2/Meg3* in pathological conditions relating to the cardiovasculature have been demonstrated. Recent studies in mice and cell culture systems suggest a role for this *Dlk1-Dio3* lncRNA in regulating stress-response in the heart, and are detailed below.

### 4.1. Gtl2/Meg3 in Cardiomyocytes

Exposing H9c2 cells, a rat cardiomyocyte-like cell line, to hypoxic conditions resulted in upregulated expression of *Gtl2/Meg3* [100]. Gong et al., showed that shRNA-mediated *Gtl2/Meg3* knockdown greatly attenuated hallmarks of hypoxic injury and restored H9c2 viability and migration. Knockdown also reduced the overall percentage of apoptotic cells and markers, and taken together, this work indicates a robust role for *Gtl2/Meg3* in facilitating hypoxic cell death Mechanistically, *Gtl2/Meg3* appears to function as a molecular decoy via its interactions with miR-183 which has been shown to target the PI3K-Akt pathway. *Gtl2/Meg3* expression levels were inversely proportional to miR-183 levels, and inhibition of miR-183 reversed the attenuation observed in *Gtl2/Meg3* knockdown H9c2 cells [102].

### 4.2. Gtl2/Meg3 in Cardiac Fibroblasts and Pressure Overload

As described previously, cardiac fibroblasts are central players in pathological myocardial remodeling. Global lncRNA profiling of myocytes and non-myocytes of the adult mouse heart revealed that *Gtl2/Meg3* is primarily enriched in cardiac fibroblasts. Piccoli and colleagues have shown that *Gtl2/Meg3* transcripts are highly enriched in adult cardiac fibroblasts. In a mouse model of chronic pressure overload *Gtl2/Meg3* transcripts were found to be significantly downregulated [103]. Curiously, further inhibition of *Gtl2/Meg3* attenuated cardiac fibrosis and decreased matrix metalloproteinase 2 (MMP2), in mice subjected to TAC [103]. Overall, *Gtl2/Meg3* appears to be an agonist of fibrosis, and its pathological downregulation in pressure overload may reflect mechanistic refinement of pathological fibrosis.

### 4.3. Gtl2/Meg3 in the Vasculature

Endothelial cell dysfunction in vasculature occurs during the human aging process, which can contribute to development of cardiovascular diseases *Gtl2/Meg3* was among to highest expressed lncRNAs in endothelial cells from human umbilical vein. It was subsequently shown that *Gtl2/Meg3* expression is higher in late passage versus early passage HUVECs, suggesting a role for this lncRNA in aging-mediated endothelial cell dysfunction. Inhibition of *Gtl2/Meg3* enhanced sprouting of aged HUVECs and improved blood flow in ischemic hind limbs of aged mice [104].

Smooth muscle proliferation plays a role in hypoxia-induced pulmonary hypertension. *Gtl2/Meg3* lncRNA is downregulated in pulmonary smooth muscle cells subjected to hypoxia [100]. Consistent with these findings, lung tissue and pulmonary arteries from patients with pulmonary arterial hypertension (PAH) displayed reduced *Gtl2/Meg3* expression [80]. Inhibition of *Gtl2/Meg3* has been shown to promote proliferation of human pulmonary artery smooth muscle cells via the PTEN and p53 pathways, respectively [100,101]. Taken together, *Gtl2/Meg3* appears to generally suppress neovascularization, and modulating *Gtl2/Meg3* activity could have utility in replenishing vasculature for diverse cardiovascular disease contexts.

## 5. Concluding Remarks

As highlighted here, the *Dlk1-Dio3* ncRNA locus has emerged as a conserved, versatile mediator of cardiovascular development and disease. In the absence of disease, it appears that *Dlk1-Dio3* ncRNAs function primarily in cardiac development, when their expression is higher compared to adult hearts (Figure 2a). While most ncRNAs in this locus are enriched during heart development, only a fraction have been functionally characterized for their specific contributions. Specifically, miRNAs expressed from this locus have been shown to influence cardiac lineage commitment (cardiomyocyte, endothelial) and embryonic/early-postnatal proliferative capacity (CPCs, fibroblasts, and cardiomyocytes). Furthermore, the observation that *Dlk1-Dio3* ncRNAs are upregulated as pluripotent stem cells transition to mesodermal-derived lineages, and subsequently differentially expressed in cardiomyocyte differentiation reinforces the notion that they have a prominent regulatory role in cardiac development (Figure 2b). As additional individual ncRNAs are investigated, we anticipate their developmental functions will become evident.

Numerous models systems have demonstrated the coordinate expression of *Dlk1-Dio3* ncRNAs, yet it remains ambiguous how these molecules–some with opposing effects–could faithfully orchestrate the proliferation, differentiation, and homeostasis of diverse cell types in the cardiovascular system. The dynamic expression patterns of *Dlk1-Dio3* ncRNAs in different phases of cardiomyocyte differentiation (Figure 2b) suggest that levels of individual ncRNAs are subject to stringent, context-specific post-transcriptional regulation. Although more precise characterization must be performed to truly understand the locus as a whole, it is tempting to speculate that *Dlk1-Dio3* ncRNA polycistron serves as an all-encompassing, but rudimentary template that can be refined to generate a complex array of functionalities.

Like many developmental regulatory factors in the heart, expression profiling strongly suggests that not only are these ncRNAs involved in cardiac development, but also function in pathological gene expression reprogramming in the diseased heart. At this juncture, it remains unclear whether pathological reactivation of locus ncRNAs is detrimental, cardioprotective, or some mixture of both. Once *Dlk1-Dio3* noncoding RNAs are understood for their dynamic roles in cardiomyocyte differentiation, proliferation and hypertrophy, this knowledge can inform medical applications aimed at improved diagnostics, therapeutics, and regenerative strategies for treatment of cardiovascular disease.

## Figures and Tables

**Table 1:** Summary of *Dlk1-Dio3* noncoding RNAs with known functions or differential expression in heart development and disease.

ncRNA	Development	Species	Disease	Species
*miRNAs*				
All *	dynamically expressed during mESC cardiomyocyte-directed differentiation [62]	m [62]		
*miR-493*	Maturation [51]	s [51]	—	
*miR-337*	—		MI [77], HF [86], angiogenesis [86], fibrosis [86], remodeling [86]. Targets STK35 [86]	h [86], m [77,86]
*miR-665*	—		CHF [92], HF [93]	h [92,93]
*miR-431*	Proliferation [15]	m [15], r [15]	—	
*miR-433*	VSD [68]	h [68]	MI [77,79], fibrosis [79], ventricular remodeling [79]. Targets AZIN1 and JNK1 [79]	m [77,79]
*miR-127*	Valve morphogenesis [66], maturation [51]	r [66], d [66], mk [66], s [51]	MI [77]	m [77]
*miR-434*	—		MI [77]	m [77]
*miR-432*	Maturation [51]	s [51]	Atrial fibrillation [99]	h [99]
*miR-136*	—		MI [77]	m [77]
*miR-370*	—		MI77, CAD [81], coronary microembolism [80]	m [77], h [81], p [80]
*miR-379*	VSD68, maturation [51]	h [68], s [51]	MI [77]	m [77]
*miR-411*	Proliferation [15], maturation [51]	m [15], r [15], s [51]	MI [77]	m [77]
*miR-299a*	—		Congestive HF [91]	h [91]
*miR-299b*	—		Congestive HF [91]	h [91]
*miR-380*	Proliferation [15], maturation [51]	m [15], r [15], s [51]	MI [95]	h [95]
*miR-323*	—		Coronary microembolism [80]	p [80]
*miR-329*	Maturation [51]	s [51]	MI77, IR-induced neovascularization [85]	m [51,85]
*miR-494*	—		I/R-induced apoptosis [89], IR-induced neovascularization [85], HF [93], arrhythmogenic RV-cardiomyopathy [96]. Targets PTEN, ROCK1, CamKIIδ, FGFR2, and LIF [89]	m [85,89], h [93,96]
*miR-1193*	—		MI [77]	m [77]
*miR-543*	Maturation [51]	s [51]	MI [77]	m [77]
*miR-495*	Proliferation [15,69], angiogenic differentiation [65]. Targets VEZF1 [65], Cited2 [69]	h [65], m [15], r [15,69]	MI neovascularization [65], fibrosis [84], MI [72,77], IR neovascularization [85], mitral valve disease [97]	h [65,84], m [72,77,85], d [97]
*miR-376c*	—		MI [77]	m [77]
*miR-654*	VSD [68]	h [68]	—	
*miR-376b*	—		MI [77]	m [77]
*miR-376a*	—		MI [77]	m [77]
*miR-300*	Differentiation [63], target of Bmi1 [63]	m [63]	—	
*miR-381*	—		Hypertrophy [73]	c [73]
*miR-487b*	VSD [68], maturation [51]	h [68], s [51]	MI [77], IR neovascularization [85]	m [77,85]
*miR-539*	Proliferation [15]	m [15], r [15]	MI [77,78], MI apoptosis [28]. Targets OGA [78] and PHB2 [28]	m [28,77,78]
*miR-544*	—		MI [77]	m [77]
*miR-382*	Maturation [51]	s [51]	MI77, comorbid aortic stenosis and CAD [98]	m [77], h [98]
*miR-134*	Proliferation [64]. Directly targets Meis2 [64]	h [64]	MI [38,94]	h [38,94]
*miR-668*	Proliferation [15], maturation [70]	m [15,70], r [15]	—	
*miR-485*	Maturation [51]	s [51]	Hypertrophy [75], MI77, remodeling [75]. Targets MAPL [75]	m [75,77]
*miR-154*	Proliferation [15], maturation [70]	m [15,70], r [15]	Hypertrophy [74], fibrosis [74,82,83], apoptosis [83]. Targets p15 [74] and DKK2 [82]	m [74,82,83]
*miR-377*	—		I/R neovascularization [86], MI neovascularization [87], fibrosis [87], Cyclosporin-mediated apoptosis [71]. Targets XIAP [71], NRP2 [71], and VEGF [87]	m [86,87], r [71]
*miR-541*	—		Hypertrophy [76], MI 77]. Repressed by MITF [76]	m [76,77]
*miR-409*	VSD [68], maturation [51]	h [68], s [51]	MI [77]	m [77]
*miR-369*	—		MI [77]	m [77]
*miR-410*	Proliferation [15,69], maturation [51]. Targets Cited2 [69]	r [15,69], m [15], s [51]	MI [72,77], I/R mitophagy [88].	m [72,77,88]
lncRNAs				
*Gtl2*	Angiogenesis [100,101]	h [100,101]	I/R apoptosis [102], fibrosis [103], hypertension [100], neovascularization [104]. Decoys miR-183 [102]	M [100,103,104], r [102]

*Key:* HF = Heart Failure, MI = Myocardial Infarction, CHF = Chronic heart failure, VSD = ventricular septal defect, CAD = coronary artery disease, RV = right-ventricular, IR = ischemia-reperfusion. For species column, h = human, m = mouse, r = rat, p = pig, s = sheep, d = dog, c = cat, mk = monkey. * All locus miRNAs, except miR-2932, -3544, -3070a, -3070b, -3071, -329, and -3072. The following miRNAs have not been reported in any publications at this time: miR-2932, miR-1906-1, miR-770, miR-673, miR-3544, miR-540, miR-3070a, miR-3070b, miR-3071, miR-341, miR-1188, miR-882, miR-1197, miR-758, miR-679, miR-666, miR-453, miR-667, miR-496a, miR-412, miR-3077, *Rtl1*, *Rian*, and *Mirg*.
